# The Debate on the Marriage Question

**Published:** 1914-04-18

**Authors:** 


### WOMEN DOCTORS AND THE LONDON COUNTY COUNCIL.

### The Debate on the Marriage Question.

In view of the interest created by the London County Council's decision to leave unchanged the rules by which women doctors in its employment must resign on marriage, we publish a summary of the debate on this question last week. This resignation-on-marriage regulation is nothing new, but several members of the Council, realising the public's interest in the matter, opposed its continuation. As we point out elsewhere, teachers in the service of the Council are exempted from it, and, in fact, are paid their usual salaries at those periods when three months' absence from duty has been necessitated as a consequence of married life.

### The Case for Non-Prohibition.

The Rev. J. Scott Lidgett moved an amendment to delete the part of the recommendation with reference to compulsory retirement on marriage.

Mr. Ed. Smith, in seconding, said this matter raised an exceedingly difficult problem, but yet one of very serious importance. He knew that there were many who said that married women could not devote the same amount of time and attention to the performance of duties as could single women; but he could say that his experience was that the best women workers were those who were married. The Census returns proved most conclusively that large numbers of nurses and teachers were married, and no one would deny that they rendered signal public service, and no fault could be found with them. The County Council had no right to impose upon these women doctors a restriction which interfered with their freedom and right to determine whether they would get married or not. A distinguished lady doctor had assured him that such a prohibition of marriage would have serious prejudicial results. In Western Australia, remarkably enough, a law had been lately passed making it a criminal offence, punishable by imprisonment or a fine not exceeding £500, for any person who brought about anything which caused a penalty to ensue to anyone getting married. It was a good thing to have married women doctors to attend the school children. After all, he liked to know that people had had personal experience.

### Equality of Treatment.

He humbly submitted that the instinct which was developed in women was a very great asset to professional ladies who had to discharge delicate duties such as these women doctors had been called upon to perform. So long as women were fully qualified, there was no reason why they should not be allowed to work, whether married or single. He recognised the point with regard to women doctors who were already in their service, but the Council could easily secure equality of treatment by making the amendment retrospective, so as to apply to all. Whilst there were some things to be argued against the employment of married women, yet it was against the spirit of the age and the best interests of the public service to enforce resignation on marriage. It was wrong and improper, and the County Council was not justified in imposing such a condition upon those in this branch of the public service. Marriage in this instance should add to the value of a woman's service and give her that experience which otherwise she would not get.

Mr. H. Gordon disagreed with these views and pointed out that the debate was really on a matter of principle and not a mere concern of the three women doctors.

### Working Wives and Low Salaries.

The principle which the Council insisted upon was that women should resign when they got married, and even the charwomen had to leave the service if they got married. They were raising not a question of individual liberty, but one of more social importance and grave economics. Where wives were encouraged to work low salaries resulted, and that was bad for the community. This was the experience in all parts of the world. There might be evils arising from restriction, but these were less than other evils.

Mr. Gray said the question was so complex that it ought not to be decided upon a side issue regarding the present appointments. The question should be considered by all the committees and reported upon fully, so that the Council could take action accordingly for all women employees.

Mr. Easton ridiculed the idea of quarrelling over such a question, in view of the extraordinary fact that the Council employed many men doctors at the mental hospitals, and at paltry salaries, and forced them to resign if they got married. If the Council proposed to allow the women doctors to marry, the men should have equal consideration. Moreover, they should be paid the same scale of salaries. Now they were not paid much better than bricklayers, but yet the Council was paying the women doctors £400 a year. Surely the mental doctors were worth as much! If they were going to make flesh of one, they could not make fish of the other. (Laughter.) His view was that when women got married their place was at home, where they should stick, and look after their families.

### A Plea for Married Doctors and Nurses.

Mr. Phillimore saw no reason why married women should be driven from the public service. They should be allowed to work. Certainly it was not fair that the London County Council should use compulsion to prevent women from getting married.

Miss Adler said valuable workers would be lost if the Council insisted on the marriage bar. The most able and experienced women would be precluded from serving London. It would be of enormous value to the Council to have married women doctors. It would, too, be wise to have married nurses. The theory that women's work reduced wages generally did not hold good in these times, when women recognised the value of co-operation and organisation.

Miss Wallas urged that if the Council desired to secure women of wide experience it would never do to dismiss them upon marriage.

Lady St. Helier, however, took a different view, urging that there were grave objections to the employment of married women. She would even like to see them dismissed from the teaching profession, for no married woman could go out to work and also do her duty to her home and family.

The amendment was rejected, thirty voting for and seventy-two against. Thus the three women doctors must resign if they marry.

